# Activity Evaluation and Mode of Action of ICA Against *Toxoplasma gondii* In Vitro

**DOI:** 10.3390/biom15020202

**Published:** 2025-02-01

**Authors:** Yanhua Qiu, Weiwei Wang, Qing Wang, Jing Xu, Guonian Dai, Yubin Bai, Jiyu Zhang

**Affiliations:** 1 Key Laboratory of New Animal Drug Project of Gansu Province, Lanzhou 730050, China; qiuqiu@nwafu.edu.cn (Y.Q.); wangweiwei@caas.cn (W.W.); 107332214028@st.gsau.edu.cn (Q.W.); 82101221334@caas.cn (J.X.); 107332214027@st.gsau.edu.cn (G.D.); 2 Key Laboratory of Veterinary Pharmaceutical Development, Ministry of Agriculture and Rural Affairs, Lanzhou 730050, China; 3 Lanzhou Institute of Husbandry and Pharmaceutical Sciences, Chinese Academy of Agricultural Sciences, Lanzhou 730050, China

**Keywords:** *Toxoplasma gondii*, ICA, ultrastructure, mitochondria

## Abstract

Toxoplasmosis is a significant zoonotic parasitic disease. Currently, there is no effective vaccine available to prevent human infection, and treatment primarily relies on chemotherapy. However, the lack of specific therapeutic agents and the limitations of existing drugs highlight the urgent need for novel, safe, and effective anti-*Toxoplasma gondii* (*T. gondii*) medications. In this study, we evaluated the toxicity of ICA (N-(pyridin-2-yl)-4-(pyridine-2-yl)thiazol-2-amine) to host cells and assessed its inhibitory and anti-proliferative effects on *T. gondii* tachyzoites. We further investigated the impact of ICA on the ultrastructure of *T. gondii* using transmission electron microscopy (TEM). Additionally, we measured alterations in mitochondrial membrane potential, superoxide levels, and ATP levels in *T. gondii* to assess the effect of ICA on mitochondrial function. Our findings demonstrated that ICA exhibits a safe and effective inhibitory effect on *T. gondii*, with a selectivity index (SI) of 258.25. Notably, ICA demonstrated a more potent anti-proliferative effect than pyrimethamine (PYR). Ultrastructural observations revealed that ICA induces mitochondrial swelling and membrane rupture in *T. gondii*. Further investigations confirmed that ICA leads to mitochondrial dysfunction in *T. gondii*. In conclusion, our results suggest that ICA possesses the potential to serve as a lead compound for the development of novel anti-*T. gondii* therapies.

## 1. Introduction

*Toxoplasma gondii (T. gondii)* is an obligate intracellular protozoan parasite belonging to the phylum Apicomplexa [1]. This organism exhibits a broad host range, capable of infecting all warm-blooded animals, including humans [2]. Numerous studies have suggested that cold-blooded animals, such as snakes and fish, and mollusks, including shellfish and snails, may also serve as potential reservoirs for *T. gondii* [3,4,5]. *T. gondii* is the causative agent of toxoplasmosis, a zoonotic disease of significant medical and veterinary importance that can be transmitted via various routes [6]. Felines are the definitive hosts for *T. gondii* and can shed millions of oocysts in their feces daily following infection; these oocysts can remain viable in the environment for several months [7]. Humans and animals can acquire *T. gondii* infection through congenital transmission, ingestion of tissue cysts present in undercooked meat, or consumption of water, soil, or food contaminated with oocysts [8]. It is estimated that approximately one-third of the global population is infected with *T. gondii* [9]. While most immunocompetent individuals remain asymptomatic, toxoplasmosis can be fatal for immunocompromised individuals and developing fetuses [8]. Additionally, certain atypical strains of *T. gondii* may cause severe symptoms or death even in immunocompetent hosts [10].

Vaccines play a crucial role in preventing toxoplasmosis in certain animal populations. However, only four commercially available *T. gondii* vaccines are currently licensed for use in sheep [11]. No vaccine is currently available for human use. Current treatment for toxoplasmosis typically involves combinations of medications, such as pyrimethamine–sulfadiazine or trimethoprim–sulfamethoxazole with antifolate drugs, spiramycin, and pyrimethamine combined with clindamycin, azithromycin, or atovaquone [12,13,14]. These therapeutic agents exhibit limited specificity, are often associated with significant adverse effects, and may not always achieve optimal efficacy [11,15]. Consequently, there is a pressing need to develop novel treatment options, particularly in the realm of chemotherapy for toxoplasmosis. The drug discovery process for *T. gondii* can generally be categorized into two main approaches: ligand-based methods, which involve repurposing existing drugs, and structure-based methods, which include rational design informed by the three-dimensional structure of the drug target [16]. Moreover, promising strategies to target multiple enzymes also have proven effective in overcoming drug resistance and improving anti-parasitic therapies [17].

**Figure 1:**
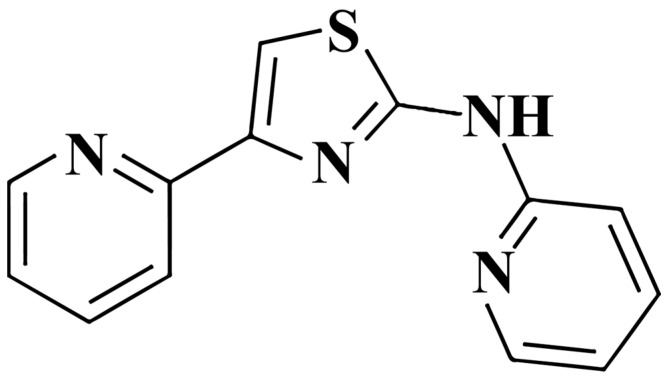
Chemical structure of ICA.

In recent years, aminothiazoles have garnered significant attention for their chemotherapeutic potential in the treatment of various diseases, including malaria [18], prion diseases [19], cancer [20], and tuberculosis [21]. ICA, or N-(pyridin-2-yl)-4-(pyridin-2-yl)thiazol-2-amine, is an aminothiazole compound (Figure 1). ICA exhibits antiarrhythmic effects [22]. It also acts as a small-conductance Ca^2+^-activated K^+^ channels inhibitor, capable of blocking SK channels by chelating cations and potentially serving as a therapeutic agent for the prevention and treatment of atrial fibrillation [23,24]. Additionally, Girardini et al. demonstrated the in vitro anti-*Leishmania* activity of ICA [25]. Based on these findings, we hypothesized that ICA might also exhibit efficacy against *T. gondii*. Therefore, this study sought to evaluate the in vitro activity of ICA against *T. gondii* and to conduct a preliminary investigation into its mode of action in combating this parasite.

## 2. Materials and Methods

### 2.1. Cytotoxicity Assay

Hs27 cells (ATCC, CRL-1634, Manassas, VA, USA) were cultured in Dulbecco’s Modified Eagle’s Medium (DMEM, Gibco, Grand Island, NY, USA) supplemented with 10% fetal bovine serum (FBS, Gibco, Grand Island, NY, USA). ICA (MCE, Monmouth Junction, NJ, USA) was added to a monolayer of Hs27 cells in 96-well plates at concentrations of 32, 16, 8, 4, 2, 1, 0.5, and 0.25 μM. The cells were then incubated in a humidified cell culture incubator maintained at 37 °C with 5.0% CO_2_ for 72 h. Following this incubation period, 10 μL of CCK-8 (MCE, Monmouth Junction, NJ, USA) reagent was added to each well, and the absorbance of each well was measured at a wavelength of 450 nm using a microplate reader (Multiskan GO, Thermo Fisher, Waltham, MA, USA) one hour later. The experiment was performed in triplicate, and GraphPad Prism (Version 9; La Jolla, CA, USA) was utilized for curve fitting to generate the dose–response curve and determine the half-maximal cytotoxic concentration (CC_50_) of ICA on Hs27 cells.

### 2.2. Inhibition Assay

RH tachyzoites expressing β-galactosidase (RH-2F, ATCC 50839, Manassas, VA, USA) were employed for growth inhibition assays. Tachyzoites were cultured in DMEM medium supplemented with 3% FBS. Fresh RH-2F tachyzoites were harvested from Hs27 cells. A total of 200 tachyzoites were inoculated into each well of a monolayer of Hs27 cells in a 96-well plate, along with various concentrations of ICA. A 0.1% DMSO solution served as the vehicle control, while 10 μM pyrimethamine (PYR) (MCE, Monmouth Junction, NJ, USA) was used as a positive control. After a 72 h incubation period, 10 μL of 10% Triton X-100 and 1 mM chlorophenol red-β-D-galactopyranoside (CPRG, Sigma, St. Louis, MO, USA) were added to each well. The incubation continued for an additional 12 h, after which the absorbance was measured at 570 nm. The experiment was performed in triplicate, and GraphPad Prism 9 software was utilized for curve fitting to generate the graph and determine the half-maximal inhibitory concentration (IC_50_) of ICA against RH-2F. The selectivity index (SI) of ICA was subsequently calculated using the formula SI = CC_50_/IC_50_.

### 2.3. Antiproliferation Assay

A total of 1 × 10^5^ RH tachyzoites were introduced to Hs27 cells in a 12-well plate. The *T. gondii* RH strain was generously provided by the Lanzhou Veterinary Research Institute, Chinese Academy of Agricultural Sciences. After a 2 h invasion period, extracellular *T. gondii* were removed by washing with PBS. Subsequently, 4 μM ICA was added, with 0.1% DMSO serving as the vehicle control and 10 μM PYR as the positive control. Cells and *T. gondii* were fixed at 24, 48, and 72 h, respectively, then permeabilized with 0.2% Triton X-100. Rabbit polyclonal to *T. gondii* (Abcam ab138698, Cambridge, MA, USA) and anti-rabbit Alexa Fluor 488 (Abcam ab150077, Cambridge, MA, USA) were used to label *T. gondii*, while nuclei were stained with DAPI (MCE, Monmouth Junction, NJ, USA). Observations and image acquisition were performed using a confocal microscope (LSM 800, Carl Zeiss AG, Oberkochen, Germany). The experiment was conducted in triplicate, with 15 randomly selected fields of view photographed at each time point in each group. Image J software (Version 1.54, National Institutes of Health, Bethesda, Rockville, MD, USA) was used to analyze the average green fluorescence intensity within each image from the respective groups.

### 2.4. Plaque Assay

Five hundred RH tachyzoites were added to a monolayer of Hs27 cells cultured in an 18 mm diameter Petri dish. After a 2 h invasion period, the cells were washed three times with PBS, then treated with either 4 μM ICA, 10 μM PYR (positive control) or 0.1% dimethyl sulfoxide (DMSO) as vehicle control. After 7 days of incubation, the cells were fixed with 4% paraformaldehyde, stained with 1% crystal violet, and subsequently photographed. The experiment was performed in triplicate.

### 2.5. Transmission Electron Microscopy Assay

RH tachyzoites were introduced to Hs27 cells in T75 flasks at a multiplicity of infection (MOI) of 2:1. Twelve hours post-infection, either 4 μM ICA or 0.1% DMSO (as a vehicle control) was added. After an additional 24 h incubation period, samples were prepared for TEM according to previously established protocols [26]. The effect of ICA on the ultrastructure of *T. gondii* was subsequently assessed using a TEM (HT7800, HITACHI, Tokyo, Japan).

### 2.6. Mitochondrial Membrane Potential Assay of Extracellular T. gondii

Five million freshly purified tachyzoites were treated with 4 μM ICA or 0.1% DMSO for 6 h. Following treatment, the samples were centrifuged to collect the *T. gondii* pellet, which was subsequently washed with PBS. Subsequently, 100 nM MitoTracker™ Red CMXRos (Thermo Fisher Waltham, MA, USA) was added for mitochondrial staining, and the samples were incubated for 30 min. After another wash with PBS, the fluorescence intensity of each group was measured using a flow cytometer (CytoFLEX LX, Beckman Coulter, CA, USA), and the mean fluorescence intensity was calculated using FlowJo™ v10.8 software (BD Biosciences, Franklin Lakes, NJ, USA). Additionally, another portion of the samples was observed and imaged under a confocal microscope.

### 2.7. Mitochondrial Superoxide Assay

Consistent with the mitochondrial membrane potential assay, extracellular tachyzoites were treated with ICA and DMSO for a duration of six hours. Subsequently, the tachyzoites were incubated with 5 μM MitoSOX Red (MCE, Monmouth Junction, NJ, USA) for 30 min at 37 °C. Following this incubation, the tachyzoites were washed with PBS, and the fluorescence intensity was measured using flow cytometry.

### 2.8. ATP Content Assay

Freshly purified *T. gondii* tachyzoites (1 × 10^7^) were incubated with ICA at concentrations of 1, 2, and 4 μM. A 0.1% DMSO solution served as the vehicle control. After 6 h, the tachyzoites were collected and washed twice with PBS, and their ATP content was measured using an enhanced ATP assay kit (Beyotime, Shanghai, China) according to the manufacturer’s instructions. The experiment was conducted in triplicate.

### 2.9. Mitochondrial Membrane Potential Assay of Hs27 Cells

Hs27 cells were seeded onto cell slides in 12-well plates and incubated with 4 μM ICA or 0.1% DMSO as a control. After 24 h, 100 nM MitoTracker^TM^ Red CMXRos (Thermo Fisher Waltham, MA, USA) was added for 30 min at 37 °C. Following a PBS wash, the cells were stained with 10 μg/mL Hoechst 33342 (MCE, USA) for 10 min. Cells on the slides were then imaged using a laser confocal microscope. This experiment was performed in triplicate. The mean fluorescence intensity of MitoTracker^TM^ Red CMXRos from nine images per condition was analyzed using ImageJ software (Version 1.54, National Institutes of Health, Bethesda, Rockville, MD, USA).

## 3. Results

### 3.1. Safety and Efficacy of ICA Against T. gondii Infection

**Figure 2:**
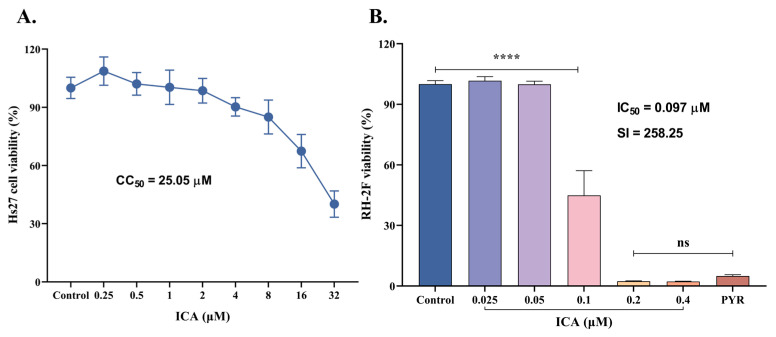
Inhibitory effects of ICA on HFF cells and *T. gondii*. Cytotoxicity of ICA on HFF cells (**A**). Inhibitory effect of ICA on RH-2F tachyzoites of *T. gondii* (**B**). Reference drug: PYR—pyrimethamine. Data are expressed as mean ± SEM. **** *p* < 0.0001, “ns” not significant, by one-way ANOVA followed by Tukey’s post hoc test.

The cytotoxicity of ICA on Hs27 cells was initially assessed over 72 h. Results demonstrated a dose-dependent cytotoxic effect of ICA on HFF cells, with a CC_50_ value of 25.05 μM. A concentration of 4 μM, corresponding to a 90.22% cell survival rate, was selected as the maximum concentration for subsequent experiments (Figure 2A). The inhibitory effect of ICA on *T. gondii* (RH-2F strain) was then evaluated. ICA exhibited significant inhibitory activity against *T. gondii*, with an IC_50_ value of 0.097 μM and a selectivity index of 258.25 (Figure 2B). Taken together, the cytotoxicity and inhibition assays suggest that ICA demonstrates both safety and efficacy against *T. gondii*.

### 3.2. ICA Suppresses T. gondii Proliferation

**Figure 3:**
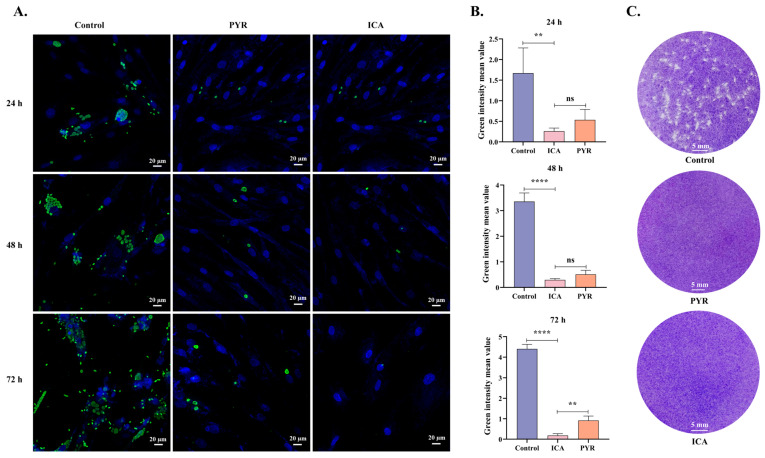
Antiproliferative effect of ICA on intracellular *T. gondii*. Cellular immunofluorescence images (**A**). Mean fluorescence intensity of green fluorescence in each image (**B**). Results of plaque assay (**C**). Reference drug: PYR—pyrimethamine. Data are expressed as mean ± SEM. ** *p* < 0.01, **** *p* < 0.0001, “ns” not significant, by one-way ANOVA followed by Tukey’s post hoc test.

*T. gondii* tachyzoites were treated with ICA following parasite invasion. At 24, 48, and 72 h, *T. gondii* and host cell nuclei were labeled with polyclonal anti-*T. gondii* antibodies and DAPI, respectively. These analyses revealed that both ICA and PYR significantly inhibited intracellular *T. gondii* proliferation (Figure 3A). Using Image J software, the mean green fluorescence intensity (representing *T. gondii*) was quantified from at least 15 immunofluorescence images per condition. The antiproliferative effect of ICA on intracellular *T. gondii* was comparable to that of PYR at 24 and 48 h, but significantly greater than that of PYR at 72 h (Figure 3B). This efficacy of ICA was further corroborated by plaque assay results, similar to PYR, which demonstrated effective protection of host cells from *T. gondii*-mediated lysis (Figure 3C). These findings suggest that ICA exhibits a more potent antiproliferative effect on *T. gondii* than PYR.

### 3.3. ICA Alters T. gondii Morphology

**Figure 4:**
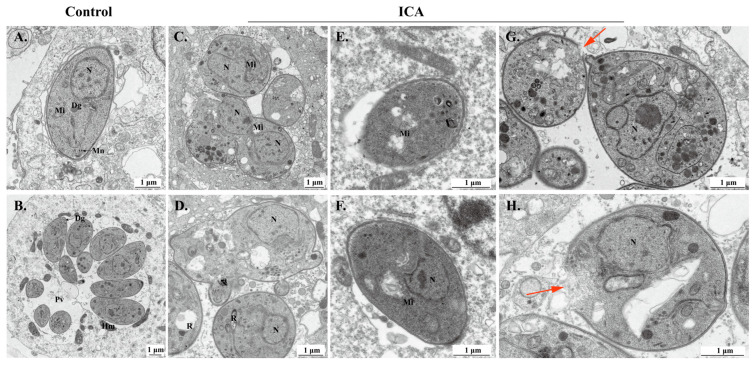
Effect of ICA on ultrastructure of *T. gondii*. Ultrastructure of normal *T. gondii* in control group (**A**,**B**). Ultrastructure of *T. gondii* treated with ICA for 24 h in cells (**C**–**H**). Red arrow points to rupture of *T. gondii* membrane. Dg, dense granule; Hm, host mitochondria; Mn, micronemes; N, nucleus; PV, parasitophorous vacuole; R, rhoptry; Mi, mitochondria.

TEM was next employed to investigate the impact of ICA on the ultrastructure of *T. gondii*. In the control group, parasites proliferated endogenously within host cells, exhibiting typical organelle ultrastructure (Figure 4A,B). Following 24 h of ICA treatment, intracellular parasites displayed significant morphological alterations compared to the control group (Figure 4C,D). Notably, mitochondria exhibited swelling and loss of cristae (Figure 4E,F). Additionally, some parasite membranes showed evidence of rupture, resulting in the release of cellular contents (Figure 4G,H).

### 3.4. ICA Causes Mitochondrial Dysfunction in T. gondii

**Figure 5:**
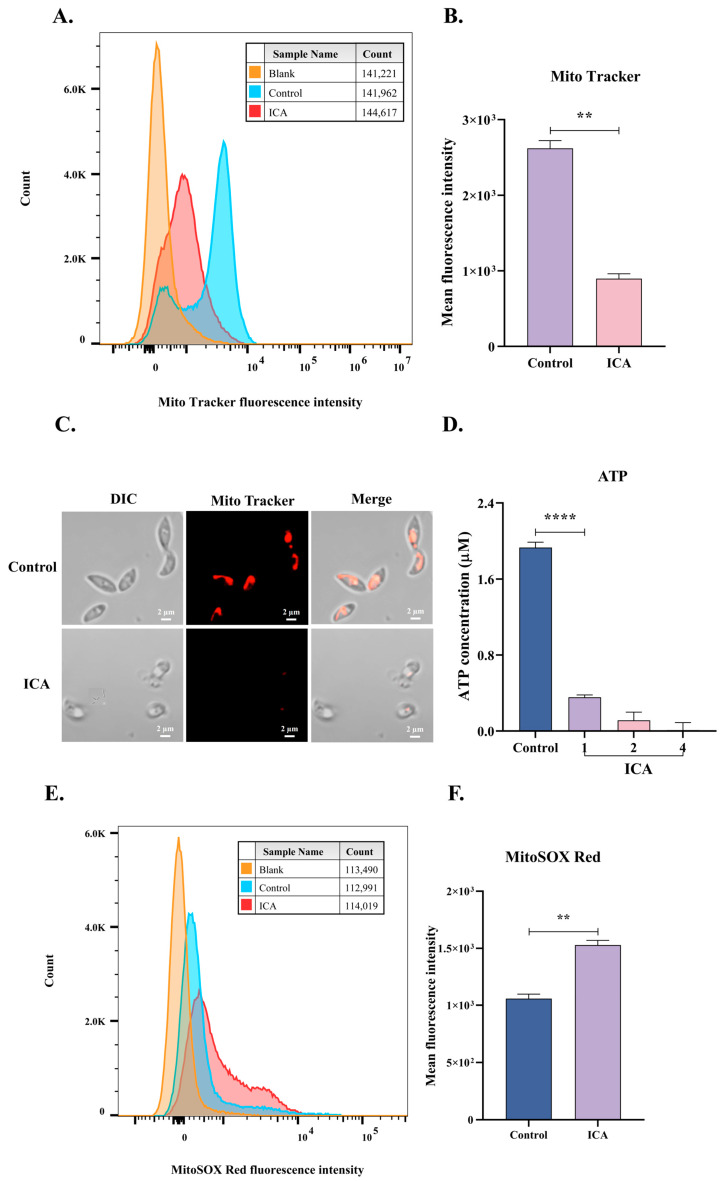
ICA causes mitochondrial dysfunction in *T. gondii*. After incubating extracellular tachyzoites with ICA for 6 h, samples were stained with MitoTracker^TM^ red CMXRos or MitoSOX Red for 30 min. Fluorescence intensity of each group was measured using flow cytometry (**A**,**E**), and mean fluorescence intensity for each group was subsequently calculated (**B**,**F**). Concurrently, mitochondrial membrane potential of *T. gondii* was assessed using laser confocal microscopy (**C**). Finally, impact of ICA on ATP levels in *T. gondii* was evaluated (**D**). Data are expressed as mean ± SEM. ** *p* < 0.01, **** *p* < 0.0001, unpaired Student’s *t*-test and one-way ANOVA followed by Tukey’s post hoc test.

Subsequently, TEM revealed mitochondrial alterations in *T. gondii*, prompting the hypothesis that ICA induces mitochondrial dysfunction in this organism. To validate this hypothesis, we employed flow cytometry to assess changes in mitochondrial membrane potential and superoxide levels in *T. gondii*, determining the mean fluorescence intensity for each experimental group. Following six hours of ICA exposure to extracellular *T. gondii*, a significant reduction in mitochondrial membrane potential was observed compared to the control group (Figure 5A,B), concurrent with a substantial increase in mitochondrial superoxide levels (Figure 5E,F). Laser confocal microscopy demonstrated that control tachyzoites maintained a normal crescent-shaped morphology, whereas ICA-treated tachyzoites exhibited morphological abnormalities and a significant decrease in mitochondrial membrane potential (Figure 5C). Finally, we evaluated the effect of ICA on *T. gondii* ATP levels, which demonstrated a significant, dose-dependent reduction (Figure 5D). These findings indicate that ICA induces mitochondrial dysfunction in *T. gondii*.

### 3.5. ICA Does Not Alter the Mitochondrial Membrane Potential of Host Cells

**Figure 6:**
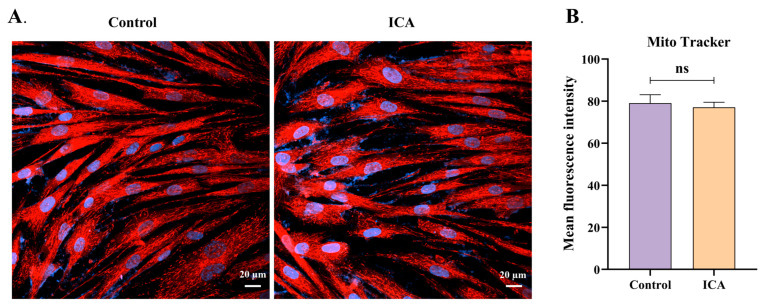
Effect of ICA on mitochondrial membrane potential in Hs27 cells. Hs27 cells were treated with ICA for 24 h, after which they were stained with MitoTracker^TM^ red CMXRos and Hoechst 33342. Stained cells were subsequently observed and photographed using a laser confocal microscope (**A**). Image J was employed to calculate mean fluorescence intensity for both control and ICA-treatment groups (**B**). Data are expressed as mean ± SEM. “ns” not significant, unpaired Student’s *t*-test.

ICA reduces the mitochondrial membrane potential of extracellular *T. gondii*; however, its effect on the host cell mitochondrial membrane potential is unclear. To investigate this, we assessed the effect of 4 μM ICA on the mitochondrial membrane potential of Hs27 cells. Our results indicate that ICA did not alter the mitochondrial membrane potential of these cells (Figure 6B). Mitochondria in both control and ICA-treated groups were observed in the cytoplasm and displayed normal morphologies, including linear, short rod-shaped, and spherical forms (Figure 6A).

## 4. Discussion

*T. gondii* exhibits a broad host range, diverse transmission routes, and a complex life cycle, classifying it as an obligate intracellular parasitic protozoan [2,27]. These characteristics present significant challenges to vaccine development, leaving chemotherapy as the primary treatment strategy for toxoplasmosis. However, currently available anti-*T. gondii* chemotherapies are limited by several drawbacks, including adverse reactions, incomplete efficacy, and low specificity [28]. Consequently, the development of novel, effective, and safe anti-*T. gondii* agents is urgently required. In this study, we report the novel finding of significant in vitro anti-*T. gondii* activity of ICA and present a preliminary investigation into its mechanism of action.

In vitro studies demonstrated that ICA exhibited significant inhibitory activity against *T. gondii* tachyzoites, effectively reducing parasite proliferation within host cells. Its anti-proliferative effects were notably superior to those of the positive control drug PYR. To investigate the mechanism of action of ICA on *T. gondii*, TEM revealed that ICA induced morphological changes and membrane rupture in the parasite. Specifically, ICA caused mitochondrial swelling and the loss of mitochondrial cristae. Mitochondria, central to cellular energy metabolism, are the primary site of ATP production [29]. Mitochondrial function is a crucial indicator of cellular health, which can be assessed by monitoring changes in mitochondrial membrane potential [30]. Additionally, mitochondrial dysfunction can lead to increased production of reactive oxygen species (ROS), resulting in oxidative stress [31]. Therefore, we evaluated the impact of ICA on *T. gondii* mitochondria by measuring mitochondrial membrane potential, superoxide levels, and ATP levels. Our results indicated that ICA induced mitochondrial dysfunction in *T. gondii*. Wescott et al. demonstrated that 2-aminothiazoles specifically target the enolase enzyme of *Mycobacterium tuberculosis*. Exposure to 2-aminothiazoles leads to the accumulation of upstream glycolytic metabolites and a reduction in ATP levels [32]. In *T. gondii*, the enolase ENO2 targets the nucleus of actively replicating parasites, specifically binding to nuclear chromatin in vivo. Disruption of the *TgENO1* gene reduced brain cyst burden in chronically infected mice and altered the transcription levels of several nuclear genes. The findings of Mouveaux et al. suggest that *T. gondii* enolase functions extend beyond glycolysis, including a direct role in coordinating gene regulation [33]. Given that ICA is a 2-aminothiazole compound, further investigation is warranted to determine whether it targets enolase in *T. gondii*.

ICA exhibits drug-like properties, with a molecular weight below 500 Da, a log *p*-value under 5, fewer than five hydrogen bond donors, and fewer than ten hydrogen bond acceptors, consistent with Lipinski’s and Veber’s rules [25]. Given its significant activity against *T. gondii*, ICA represents a promising lead compound for targeting this pathogen.

## 5. Conclusions

Overall, our results demonstrate that ICA exhibits significant and effective inhibitory activity against *T. gondii*, significantly reducing parasite proliferation within host cells. Ultrastructural analysis revealed that ICA induces morphological alterations in tachyzoites, including mitochondrial swelling and plasma membrane rupture. Further investigation indicated that ICA induces mitochondrial dysfunction in *T. gondii* while sparing host cell mitochondria. These findings suggest that ICA has potential as an effective anti-*T. gondii* therapeutic agent.

## Data Availability

The original contributions presented in this study are included in the article. Further inquiries can be directed to the corresponding authors.
